# A retrospective cohort report of single-incision laparoscopic cholecystectomies in Saudi Arabia: Postoperative outcomes and patient satisfaction

**DOI:** 10.1016/j.amsu.2022.104245

**Published:** 2022-07-31

**Authors:** Khalil Terro, Mohanad Baroudi, Rawan Alsaoud, Belal Nedal Sabbah, Ahmed Abunimer, Salim Abduljawad, Saud Al-Shanafey

**Affiliations:** a Department of Surgery, Specialized Medical Center Hospital, Riyadh, Saudi Arabia; b College of Medicine, Alfaisal University, Riyadh, Saudi Arabia; c Department of Surgery, King Faisal Specialist Hospital & Research Centre, Riyadh, Saudi Arabia

**Keywords:** Cholecystectomy, Single-incision, Laparoscopic, Minimally invasive

## Abstract

**Introduction:**

Single incision laparoscopic cholecystectomy has become more popular recently. Because it yields shorter hospitalization, less postoperative pain, and better cosmetic outcomes. As it minimizes the number of incisions, it causes less trauma to the anterior abdominal wall and this decreases the operative mortality and morbidity. In this study, our aim is to share our results from the procedure so that surgeons in the field may consider adopting this approach when performing a laparoscopic cholecystectomy.

**Methods:**

This is a retrospective study of 125 patients that underwent single-incision cholecystectomy. These patients underwent the procedure in a specialized center. We extracted data and surveyed patients who underwent the procedure between 2017 and 2019, and that were performed by the same consultant using the standard tools of laparoscopic surgery. All patients were followed for 12 months. The postoperative survey includes; the cosmetic appearance of the surgical site, pain management after the procedure, and patient satisfaction with this experience.

**Results:**

Most of the patients were satisfied with postoperative pain management and their cosmetic appearance. Most of the patients were females diagnosed with cholelithiasis preoperatively. The mean age of the patients was 37.43 ± 10.72 years, the mean BMI of the participants was 29.68 ± 6.51 kg/m^2^ and the mean operative time was 25.56 ± 10.42 min.

**Conclusion:**

Single incision laparoscopic cholecystectomy has the potential to become the procedure of choice for cholecystectomy.

## Highlights

• Single incision laparoscopic cholecystectomy has become more popular recently. Because it yields shorter hospitalization, less postoperative pain, and better cosmetic outcomes.
• Single incision laparoscopic cholecystectomy has shown exceptional post outcome patient satisfaction, and reduced operative time.
• Single incision laparoscopic cholecystectomy might soon become the procedure of choice for cholecystectomy. .

## 1 Introduction

Minimally invasive cholecystectomy in the form of single-incision laparoscopy has become increasingly popular in recent years. A considerable number of studies in various medical centers have concluded that single-incision laparoscopic cholecystectomy (SILC) results in shorter hospitalization, less postoperative pain, and better cosmetic outcomes [1,2]. Since laparoscopic cholecystectomy is one of the most commonly performed procedures, it is important to refine its quality and safety to improve patient satisfaction. SILC is an emerging and promising approach that aims to minimize the number of incisions, reduce abdominal wall and skin trauma, and reduce the incidence of perioperative morbidity and mortality [3]. Although this procedure is commonly performed using expensive specialized equipment, we report here our experience and surgical results with single-incision laparoscopy in the treatment of different pathologies using standard laparoscopic tools. In this study, our aim is to share our results from the procedure so that surgeons in the field may consider adopting this approach when performing a laparoscopic cholecystectomy.

## 2 Methods

This is a retrospective cohort study for patients who underwent cholecystectomy in a specialized center between 2017 and 2019. Institutional review board approval was waived for the study. This study is reported in line with the STROCSS criteria [4]. Research Registry Unique Identifying Number: researchregistry8115.

### 2.1 Patient selection

All patients undergoing single-incision laparoscopic cholecystectomy were eligible for the study. The inclusion criteria included all patients with sufficient demographic and follow-up data. Patients with missing data or a follow-up period of less than 12 months were excluded from the analysis. Patient data was collected from the SMC electronic medical record system. These include age, sex, body mass index (BMI), presenting complaints, diagnosis, operative time, operative blood loss and perioperative complications (wound infection, bleeding, and atelectasis).

### 2.2 Surgical technique

After obtaining anesthesia clearance, all patients were kept NPO for 6–8 h before the operation. All surgeries were performed under general anesthesia. In addition, the incision site was shaved and sterilized with a chlorhexidine-based or alcohol-based povidone-iodine solution.

A standard surgical technique was applied for all procedures using standard laparoscopic instruments (Storz). The abdomen was accessed through a 2 cm supraumbilical incision. After abdominal insufflation using the Veress pneumoperitoneum needle, three 5 mm ports (Covidien) were inserted through the incision. In 23 of the patients, the Veress needle was inserted into the right upper quadrant through a 2 mm incision to facilitate liver retraction. Dissection was started in the neck of the gallbladder until a critical view of safety was achieved. 5 mm clips were used to ligate the cystic duct and cystic artery. The gallbladder was then extracted through the right port after being dissected from the liver bed. Following successful extraction, hemostasis was secured. CO2 was released. Finally, the skin is closed using 4-0 Monocryl subcuticular suture. The dressing was done with steristreps and waterproof dressing.

Postoperatively, paracetamol was used for pain management in all patients. Opioids were used in cases of severe pain. The patients were kept in NPO until flatus was passed, after which clear liquid was started. A low-fat diet was administered before the patients were discharged.

### 2.3 Patient satisfaction

During the follow-up in the clinic for a period of 2 weeks and one month after operative, the patients completed a short survey. The elements of the survey are: 1) cosmetic appearance of the incision site; 2) postoperative pain; 3) general patient satisfaction. For each of these elements, a satisfaction scale (1 = very dissatisfied, 2 = dissatisfied, 3 = neutral, 4 = satisfied, 5 = very satisfied) is provided.

### 2.4 Statistical analysis

The data was analyzed using Microsoft Excel. Central tendency measures and variance measures were calculated for the different parameters that were analyzed.

## 3 Results

After excluding patients with insufficient data, 125 patients who underwent SILC were included in the analysis (Table 1). All patients were followed up for 12 months. The mean age of the patients was 37.43 ± 10.72 years. The majority of the study population were females (n = 95), and the rest were males (n = 30). The mean BMI of the participants was 29.68 ± 6.51 kg/m^2^. Of the 125 patients, 114 patients were diagnosed with cholelithiasis, 9 patients with acute calculous cholecystitis, and 2 patients with hydrops of the gallbladder. Most of the patients (80%) presented typical symptoms of biliary colic, mainly pain in the pain in the right upper quadrant exacerbated by fatty food intake. The mean operative time was 25.56 ± 10.42 min, with a maximum operating time of 65 min and a minimum of 12 min. The estimated blood loss was minimal in all operations. The rate of postoperative complications was 0%.

**Table 1:** Demographics for 125 patients undergoing single-incision laparoscopic cholecystectomy.

Variables	Measurement
Age in years, mean ± SD (median)	37.4 ± 10.7 (36)
BMI, mean ± SD (kg/m2)	29.7 ± 6.5
Operative time in minutes, mean ± SD (median)	24.6 ± 10.4 (19)
Length of stay in days, mean ± SD	1.3 ± 0.5

The results of the satisfaction survey are shown in Table 2. Sixty seven patients (53.6%) reported that they were ‘very satisfied’ with the overall experience. 53 patients (42.4%) reported that they were ‘satisfied’ with the overall experience. 4 patients (3.2%) reported a neutral level of satisfaction and only 1 patient (0.8%) was dissatisfied due to hypertrophic scar formation.

**Table 2:** The results of patients' satisfaction survey.

Level of Satisfaction	Cosmetic appearance	Postoperative pain	Overall satisfaction
Very Dissatisfied	0	1	0
Dissatisfied	2	8	1
Neutral	3	12	4
Satisfied	60	40	53
Very Satisfied	60	64	67

## 4 Discussion

SILC is being utilized in increasing numbers due to feasibility, safety, and improved patient outcomes, and this was illustrated in a good number of case series [3,5] [[3], [5], [6], [7], [8]] [[3], [5], [6], [7], [8]]. The improved safety and patient outcomes can be explained by the reduced rate of complications, as well as the overall patient experience improved in terms of pain control and cosmetic outlook. Although this procedure is not the standard of cholecystectomy, current literature and our experience indicate that SILC has the potential to be the operation of choice in the upcoming years. Shaikh et al.^8^ reported 50 cases who were operated using SILC with minimal blood loss and no postoperative complications. Similarly, Ersin et al. [6] successfully operated on 19 cases of SILC, with no complications or deaths associated with this technique.

The operative technique we followed has been described in other studies in which SILC was successfully performed using conventional laparoscopic instruments [7]. A noticeable advantage of this is that surgical costs can be significantly reduced. Henrisken et al. [1]. found that the cost of the single-incision procedure was significantly lower than that for the 4-port cholecystectomy (P < 0.0005). The fact that most surgical centers are equipped with conventional laparoscopic instruments favors the use of SILC for the purpose of economic efficiency.

One of the main variables that determines operational efficiency is operative time. As operative time decreases, the surgeon's and hospital performance capacity will increase. Therefore, our study considers the tangible reduction in operation time as an independent factor that favors SILC. Interestingly, the mean operative time in our study was 24.6 min, while other studies have reported a mean operative time ranging from 47.31 to 93.16 min with conventional laparoscopic cholecystectomy [9,10]. Although many cohorts reported the need for conversion to multi-incision or open procedure in a minor population of patients [1,5,6,11], none of our patients required such conversions.

Regarding postoperative patient satisfaction, our study shows that SILC is an excellent alternative to conventional laparoscopy. The cosmetic outcome, which is the patient's perception of their body image after the procedure, points to higher satisfaction rates. This is consistent with several other studies that have also reported a better cosmetic outcome in SILC compared to conventional laparoscopy [12,13].

As pain and wound infection are significant causes of patient morbidity, results from various studies corroborate our findings of less postoperative pain and less incidence of surgical wound infection [11]. In our study, only one patient developed wound infection after the operation. Additionally, most of our patients were comfortable with paracetamol for pain control; only 4 of them had more severe pain that was adequately managed with low doses of opioids.

One limitation of this study is that it does not compare SILC with any other surgical technique, and that we only demonstrated our criteria. Therefore, it cannot be concluded that this technique is the gold standard of treatment. This necessitates the need of further studies to be done on the procedure, so that practicing surgeons may have an improved scope of what to employe in their practice.

## 5 Conclusions

The field of minimally invasive surgery is expanding rapidly, and in order to steer these expansive efforts in the appropriate direction, the safety and satisfaction of patients should be of the utmost importance. In this study, we have demonstrated that the use of SILC is in fact an optimal, minimally invasive, and promising procedure that will greatly add on to this field. The pain control and cosmetic appearance of the patients in our study exceeded our expectations, as the level of satisfaction obtained was truly rewarding.

## Ethical approval

Specialized Medical Center (SMC) waived IRB approval for this study.

## Please state any sources of funding for your research

No funding was provided.

## Author contribution

K.T., M.B., R.A., B.N.S., A.A., S.A., S.A.S. drafted the manuscript K.T. contributed to reviewing and finalizing the manuscript. All authors reviewed the manuscript for intellectual content and approved the submission.

## Please state any conflicts of interest

The authors declare no conflicts of interest.

## Registration of research studies

1. Name of the registry:
2. Unique Identifying number or registration ID:
3. Hyperlink to your specific registration (must be publicly accessible and will be checked):

N/A.

## Guarantor

Khalil Terro, MD.

## Consent

N/A.

## Research registration

N/A.

## Provenance and peer review

Not commissioned, externally peer reviewed.

## Availability of data and material

Not applicable.

## Code availability

Not applicable.

## Consent to participate

Not applicable.
